# Supplementary figures and images for: TriatoDex, an electronic identification key to the Triatominae (Hemiptera: Reduviidae), vectors of Chagas disease: Development, description, and performance
Source: PLoS One. 2021 Apr 22;16(4):e0248628. doi: 10.1371/journal.pone.0248628 (PMC8061935; doi:10.1371/journal.pone.0248628)

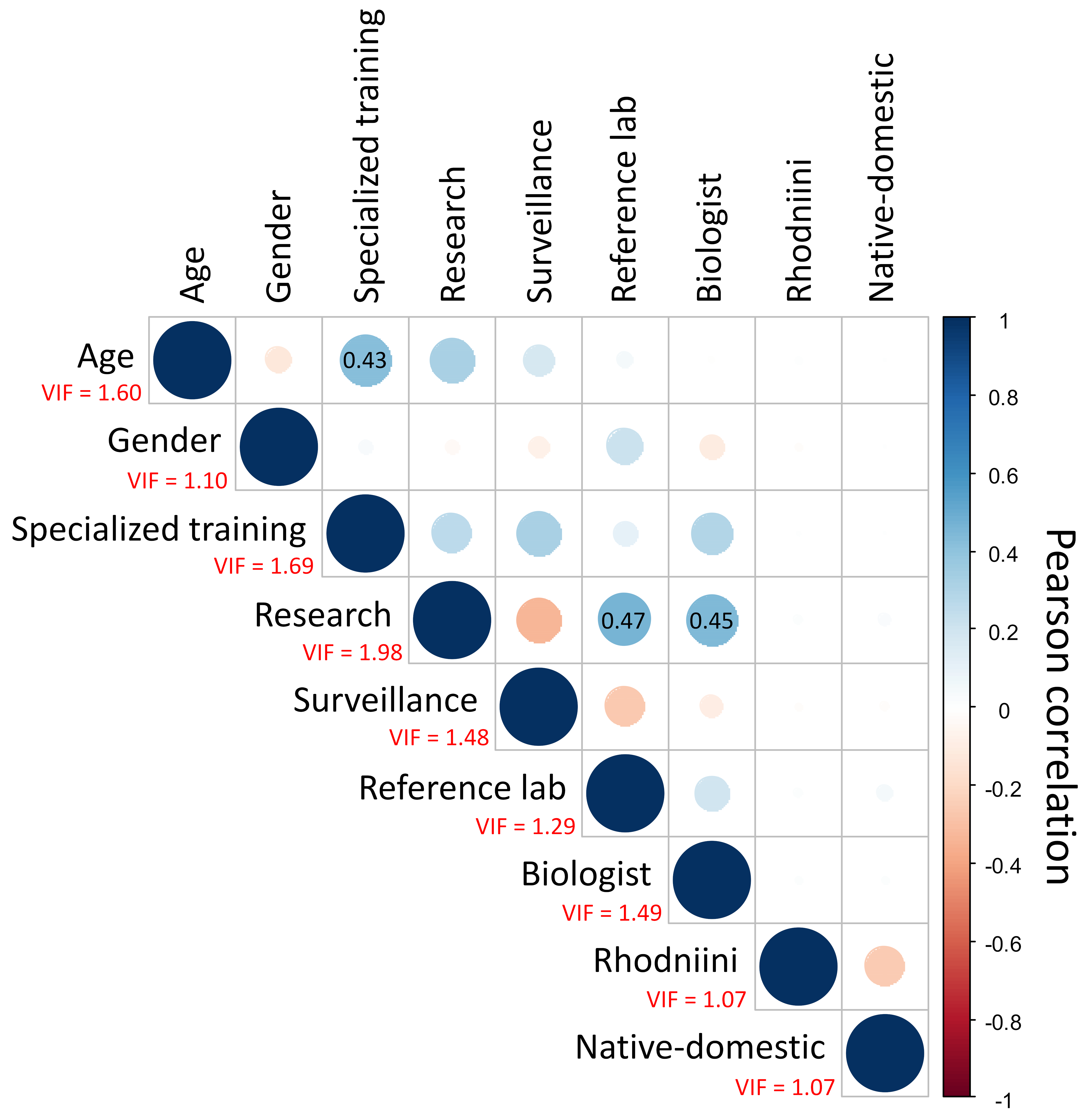

Supplement: S1 Fig — Note that correlations were all ≤ |0.47| and VIFs were all < 2.0. (TIF) [file pone.0248628.s001.tif]

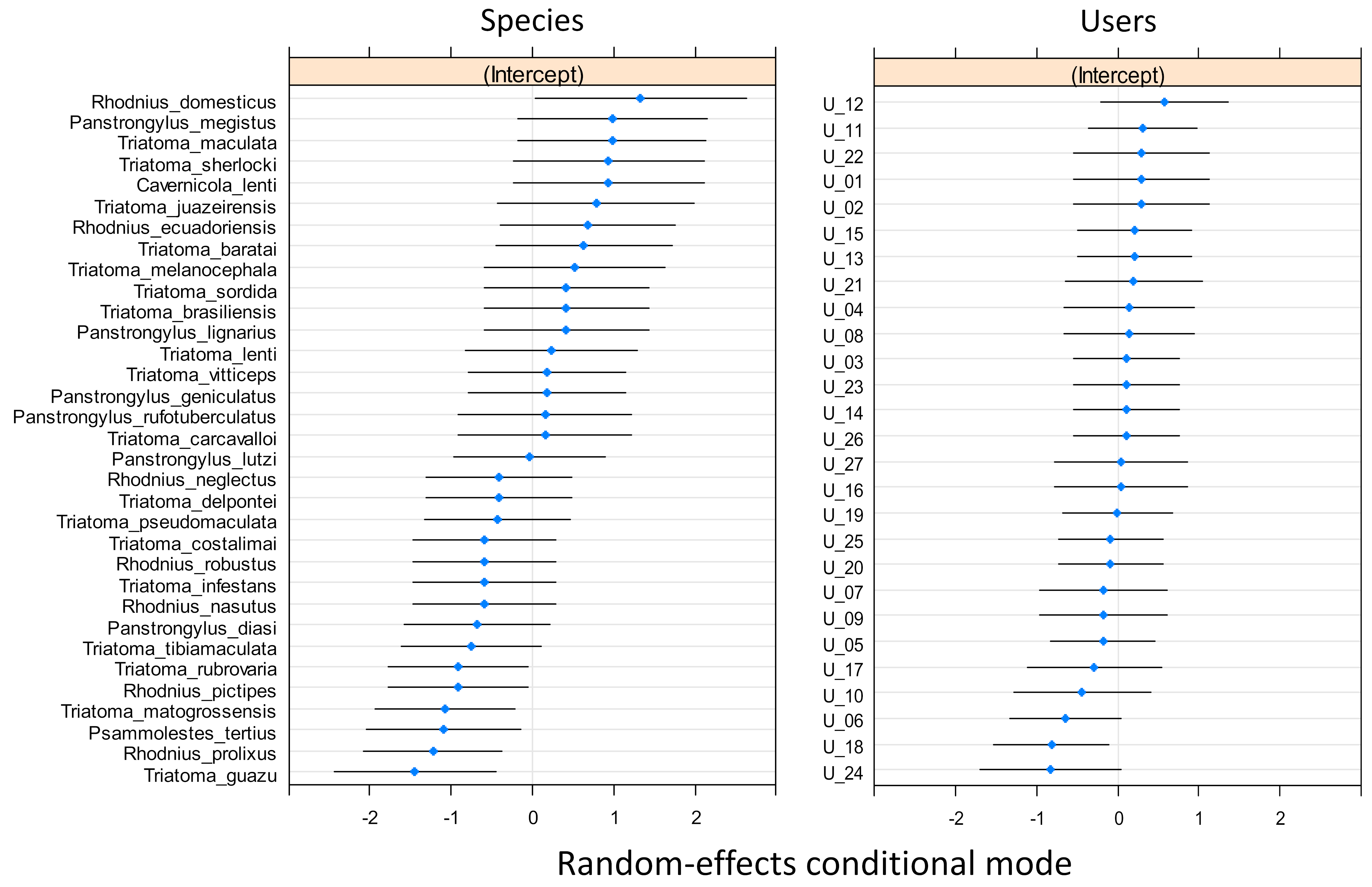

Supplement: S2 Fig — Note that random variation in TriatoDex performance was substantially larger among species than among users; the CIs spanned only negative values for six bug species but only for one user. (TIF) [file pone.0248628.s002.tif]
