# Supplementary material for: TriatoDex, an electronic identification key to the Triatominae (Hemiptera: Reduviidae), vectors of Chagas disease: Development, description, and performance
Source: PLoS One. 2021 Apr 22;16(4):e0248628. doi: 10.1371/journal.pone.0248628 (PMC8061935; doi:10.1371/journal.pone.0248628)
Supplement: S2 Table — (PDF) [file pone.0248628.s004.pdf]

S2 Table. Top-performing model (binomial, logit link-function) of TRIATODEX performance: parameter estimates, standard errors, and 95% confidence interval limits

Effects	Term	Estimate	SE	CI lower	CI upper
Fixed	Intercept	0.978	0.242	0.503	1.452
	Training	1.649	0.293	1.075	2.223
Random (SD)	User ID	0.498	-	0.285	0.869
	Species ID	0.915	-	0.629	1.333

SE, standard error; CI lower and CI upper, lower and upper limits of the 95% confidence interval; SD, standard deviation
