# Supplementary material for: TriatoDex, an electronic identification key to the Triatominae (Hemiptera: Reduviidae), vectors of Chagas disease: Development, description, and performance
Source: PLoS One. 2021 Apr 22;16(4):e0248628. doi: 10.1371/journal.pone.0248628 (PMC8061935; doi:10.1371/journal.pone.0248628)
Supplement: S3 Table — (PDF) [file pone.0248628.s005.pdf]

S3 Table. Top-performing time model (zero-truncated negative binomial, log link-function) of TRIATODEX performance in comparison with a printed key [2]: parameter estimates, standard errors, and 95% confidence interval limits

Effects	Term	Estimate	SE	CI lower	CI upper
Fixed	Intercept	1.95	0.104	1.747	2.154
	TRIATODEX	−0.398	0.058	−0.512	−0.284
Random (SD)	User ID	0.2	-	0.108	0.369
	Species ID	0.32	-	0.231	0.444

SE, standard error; CI lower and CI upper, lower and upper limits of the 95% confidence interval; SD, standard deviation
