# Supplementary material for: TriatoDex, an electronic identification key to the Triatominae (Hemiptera: Reduviidae), vectors of Chagas disease: Development, description, and performance
Source: PLoS One. 2021 Apr 22;16(4):e0248628. doi: 10.1371/journal.pone.0248628 (PMC8061935; doi:10.1371/journal.pone.0248628)
Supplement: Attachment — Submitted filename: Rebuttal letter.docx [file pone.0248628.s014.docx]

Reviewers' comments:

1. Is the manuscript technically sound, and do the data support the conclusions?

The manuscript must describe a technically sound piece of scientific research with data that supports the conclusions. Experiments must have been conducted rigorously, with appropriate controls, replication, and sample sizes. The conclusions must be drawn appropriately based on the data presented.

Reviewer #1: Yes

Reviewer #2: Yes

2. Has the statistical analysis been performed appropriately and rigorously?

Reviewer #1: I Don't Know

Reviewer #2: Yes

3. Have the authors made all data underlying the findings in their manuscript fully available?

The PLOS Data policy requires authors to make all data underlying the findings described in their manuscript fully available without restriction, with rare exception (please refer to the Data Availability Statement in the manuscript PDF file). The data should be provided as part of the manuscript or its supporting information, or deposited to a public repository. For example, in addition to summary statistics, the data points behind means, medians and variance measures should be available. If there are restrictions on publicly sharing data—e.g. participant privacy or use of data from a third party—those must be specified.

Reviewer #1: Yes

Reviewer #2: Yes

4. Is the manuscript presented in an intelligible fashion and written in standard English?

PLOS ONE does not copyedit accepted manuscripts, so the language in submitted articles must be clear, correct, and unambiguous. Any typographical or grammatical errors should be corrected at revision, so please note any specific errors here.

Reviewer #1: Yes

Reviewer #2: Yes

5. Review Comments to the Author

Please use the space provided to explain your answers to the questions above. You may also include additional comments for the author, including concerns about dual publication, research ethics, or publication ethics. (Please upload your review as an attachment if it exceeds 20,000 characters)

Reviewer #1: Review Plos Triatodex

I have a few doubts on the importance of this manuscript, as listed below:

1-The manuscript brings the analysis of a very important technological tool for the Triatominae identification and the detailed statistical analysis checking the efficacy of the Triatodex App. Despite the nice presentation of the manuscript, I am not sure it is in the scope or interesting to be published in Plos one. Therefore, I am going to decline of presenting the final decision which I leave with the editorial team.

We thank the Reviewer for her/his positive comments. As per PLOS ONE’s guidelines, available at https://journals.plos.org/plosone/static/publish and more specifically at https://journals.plos.org/plosone/s/submission-guidelines#loc-methods-software-databases-and-tools, we are confident that our submission is within the scope of the journal and meets its publication criteria (https://journals.plos.org/plosone/s/journal-information#loc-criteria-for-publication).

2-Another point is that the tool structure, tutorial, and the program itself are available on the internet since 2017 and I am not sure if this manuscript is going to complement any previous information already available in the web since 2017.

We provide a detailed description of the app and a rigorous evaluation of its performance. We also present the current limitations of our tool and outline some key perspectives for further development and improvement. We believe that sharing these topics with the broader community is important and falls well within the scope of PLOS ONE. Specifically, the journal welcomes “[S]ubmissions describing methods, software, databases, or other tools that meet the journal’s criteria for utility, validation and availability.” We believe our work fits that description and fulfils all three criteria (see https://journals.plos.org/plosone/s/submission-guidelines#loc-methods-software-databases-and-tools).

3-How important is the publication of the statistical analysis on the efficacy of the Triatominae identification tool? Its importance and efficiency are going to be proved or disapproved by the users themselves, assessing and using the App. Again, the final decision I am going to leave with the editors.

We note again that PLOS ONE’s guidelines state that “[S]ubmissions presenting methods, software, databases, or tools must demonstrate that the new tool achieves its intended purpose. If similar options already exist, the submitted manuscript must demonstrate that the new tool is an improvement over existing options in some way. This requirement may be met by including a proof-of-principle experiment or analysis; if this is not possible, a discussion of the possible applications and some preliminary analysis may be sufficient.” Our assessment of TriatoDex performance, including a comparison with a standard printed key, addresses this requirement.

Please check the following links presenting the Triatodex in the internet since 2017

https://www.youtube.com/watch?v=8-M55EoWjg0

https://www.youtube.com/watch?v=8jqGNnkU5eU

https://blogdeparasitologia.wordpress.com/2017/09/22/aplicativo-facilita-a-identificacao-morfologica-de-triatomineos/

We see our manuscript as a technical/scientific counterpart to the non-technical information these links provide access to. If our tool is to be used in disease-vector surveillance, the decision to do so will likely be based on the data and analyses we present in this manuscript.

Finaly, this manuscript does not bring complementary information or bring crucial updates related to the App. Several other papers on Triatominae systematics and taxonomy could be included to enrich the knowledge of the more than 150 species now.

Please see our previous comments. We have tried to reasonably cover the vast literature on triatomine-bug systematics; our emphasis on taxonomic novelty (refs. [36-38,49-56]) is, we believe, suitably counterbalanced by the inclusion of both classic (e.g., refs. [1,34]) and more recent in-depth reviews (e.g., refs. [2,16]). We note that we already have 67 refs.

Some suggestions are listed below:

-Title should include further information such as: the Trypanosoma cruzi vectors, Hemiptera, Heteroptera, Reduviidae, Triatominae

Perhaps “TriatoDex, an electronic identification key to the Triatominae (Hemiptera: Reduviidae), vectors of Chagas disease: development, description, and performance”

-Additional references on Triatominae systematics and taxonomy should be added in order to complement the visual and summarized information for each of the listed species in the App.

Why just Lent and Wygodzinsky, Carcavalo et al and Galvão & Dale were mentioned? And about the extensive bibliography on the species complexes? Phenotipic variations? Why these papers were not mentioned and listed?

Please see our comment above. We note that those three refs. include the most widely-used printed *identification* *keys* to the Triatominae; that is why we specifically mention them.

Reviewer #2: The authors present a novel and useful digital tool for Triatomine species identification, the vectors of Trypanosoma cruzi, a parasite that causes Chagas Disease. TriatoDex is a cell phone app currently available for android phones, which will be available also for iPhones. The authors also performed several tests to assess the performance of TriatoDex in different conditions, such as User training background, user age, insect identification difficulty, etc., in a hypothesis testing framing.

Overall, I think this is a very nice project (TriatoDex), which has been creatively evaluated under different situations and compared to other ways to identify species, such as traditional keys or automated approaches. Triatodex outperformed in several of such challenges.

I can realize this project is the result of hard work, possibly of many years. I can testify the author's group includes well-established experts in Triatomines. I encourage authors to keep TriatoDex updated and expanded when more resources and applications emerge. I appreciate the Prospect section, where authors envisage ways to improve app performance.

We thank the Reviewer for these positive remarks.

My only concern about the models is that the user ID was used as a random variable. Still, in my opinion, tasks are nested in users (i.e., identification tasks are not independent observations). If true, a nested modeling approach could be more suitable for this data.

The Reviewer is right – the results of tasks performed by the same user cannot be treated as independent. Note, however, that there is only one ‘lower level’ below the ‘higher level’ represented by users – *observations* nested within users are the lowest possible level of the hierarchy. The structure of our models captures this hierarchy, but cannot go below its lowest level. That would be different if, for example, each user had undertaken several identification tasks involving bugs of *the same species*; then, one could argue that the problem should be represented by a three-level hierarchy with observations nested within species nested within users – which could perhaps be written as {user[species(outcomes)]}. In our study, each user had to identify just one bug of each species, so the hierarchy was simply [user(outcomes)] and the models capture this structure.

Minor points:

Line 5.- Consider replacing "Triatominae" with "Insect vectors."

We prefer to use Triatominae; “insect vectors” sounds too general.

Line 10-.- Add "worldwide" before 150

Changed to “…the 150 triatomine-bug species described worldwide…”

Line 28. Any clue to the next steps to improve the reliance of TriatoDex to be used by not trained users?

We have already 301 words in the Abstract; hurdles and ways for improvement are discussed in some detail within the main body of the manuscript, particularly in the ‘Caveats’ and ‘Prospects’ sections. The last sentences of the ‘Prospects’ section specifically address the extension of TriatoDex use to “… community-health agents and other primary healthcare staff…” and “… the broader community…”

Line 51.- It may be of help to better understand the Chagas disease dimensions, adding a few reasons of why Chagas disease is the main parasitic disease in Latin America

We have changed this to “… one of the major neglected tropical diseases …” The next paragraph presents recent estimates showing high prevalence, with millions infected, and high incidence, with thousands of new infections occurring each year.

Line 76: Rephrase to emphasize that some regions are species-rich, challenging vector control measures.

Perhaps “Particularly in species-rich regions, these native triatomines pose a dual challenge to Chagas disease surveillance”

Line 85: Consider to add "worldwide" to make clear that TriatDex deals with the worldwide species diversity instead of regional coverage

Changed to “… covers the 150 triatomine-bug species formally described worldwide up to 2017…”

Line 200: Please explain in more detail why 511 models

We have 9 variables; the number of possible additive combinations of those variables is 2^9^ = 512, so we have the full model plus the “… 511 additive models nested within [that] full model…”.
